# Immersive virtual reality interferes with default head–trunk coordination strategies in young children

**DOI:** 10.1038/s41598-021-96866-8

**Published:** 2021-09-27

**Authors:** Jenifer Miehlbradt, Luigi F. Cuturi, Silvia Zanchi, Monica Gori, Silvestro Micera

**Affiliations:** 1 Bertarelli Foundation Chair in Translational Neuroengineering, Center for Neuroprosthetics, École Polytechnique Fédérale de Lausanne, 1202 Geneva, Switzerland; 2 Brain Electrophysiology Attention Movement Laboratory, Institute of Psychology, Université de Lausanne, 1015 Lausanne, Switzerland; 3 Unit for Visually Impaired People, Center for Human Technologies, Fondazione Istituto Italiano di Tecnologia, 16152 Genova, Italy; 4 Robotics Brain and Cognitive Sciences, Center for Human Technologies, Fondazione Istituto Italiano di Tecnologia, 16152 Genova, Italy; 5 DIBRIS Department, Università di Genova, 16145 Genova, Italy; 6 The Biorobotics Institute and Department of Excellence in Robotics and AI, Scuola Superiore Sant’Anna, 56025 Pontedera, Italy

**Keywords:** Motor control, Sensorimotor processing, Biomedical engineering, Human behaviour

## Abstract

The acquisition of postural control is an elaborate process, which relies on the balanced integration of multisensory inputs. Current models suggest that young children rely on an ‘en-block’ control of their upper body before sequentially acquiring a segmental control around the age of 7, and that they resort to the former strategy under challenging conditions. While recent works suggest that a virtual sensory environment alters visuomotor integration in healthy adults, little is known about the effects on younger individuals. Here we show that this default coordination pattern is disrupted by an immersive virtual reality framework where a steering role is assigned to the trunk, which causes 6- to 8-year-olds to employ an ill-adapted segmental strategy. These results provide an alternate trajectory of motor development and emphasize the immaturity of postural control at these ages.

## Introduction

Coordinated motor behavior and efficient integration of stimuli from different sensory modalities are necessary for successful interactions with the surrounding environment^1^. The development of these abilities follows a long-lasting and elaborate process, starting long before birth and extending into early adulthood. At the motor development level, the skills are usually grouped into two categories. First, gross motor skills comprise postural control and locomotion and require the use of axial and proximal muscles. The maturation of these abilities shows a steep increase until the age of 2 years and continues to refine until later childhood^2–5^. Conversely, fine motor skills include precise actions such as functional hand movements, but also require multisensory integration such as hand-eye coordination. The time course of fine motor development typically extends over a more extended time period and adult patterns are generally not observed before late childhood^6,7^.

The acquisition of a steady posture is a prerequisite for goal-directed behaviors such as reaching from a sitting position or locomotion^1,6^. According to the ontogenetic model of postural development during childhood described by Assaiante et al., two main principles guide the selection of a given balance strategy: the choice of a stable reference, which shifts from the pelvis to the head^1,8^, and the gradual mastery of the involved degrees of freedom (DOF)^1,9,10^. The coordination strategy evolves from an ‘en-block’ behavior, which minimizes the number of DOF to be controlled^11,12^ to a fully articulated strategy, where each DOF is controlled individually. Mature, multi-jointed patterns are acquired at different ages, depending on the involved joint and task characteristics. During locomotion, the ‘en-block’ stabilization has been observed from the acquisition of an upright stance until 6 years, while children aged 7 and older started to display a segmental control^10^. Similarly, rigid forearm-trunk coupling was observed until 6 years both during voluntary trunk movements and in response to trunk perturbations^13^. Instead, in a reaching task, adult head–trunk–arm coordination patterns were observed in children as young as 2–3 years old for movements in the pitch plane and from 4 years onwards in the roll and yaw planes^14^. Yet, the activity and temporal recruitment of postural muscles appear to reach mature levels only after the age of 11^8^. The ability to decouple head and trunk movements proves to be particularly useful when having to avoid or circumvent an obstacle while walking, where anticipatory head movements were observed from 5.5 years onwards, while younger children displayed a rigid head–trunk connection^15^. Children thus first build a repertoire of postural strategies, before learning how and when to adequately implement them.

Nevertheless, successful postural stabilization does not only involve appropriate multi-jointed coordination but also requires the integration of the information provided by different sensory modalities. The Bayesian model of multisensory integration suggests that adults fuse redundant sensory inputs in a statistically optimal way by weighting the sources according to their uncertainty^16,17^. The ability to combine different cues to obtain more precise estimates of one's surroundings appears late in childhood development^18,19^, that is, after the individual modalities have matured^20,21^, unless additional feedback on the reliability of each cue is provided^22^. Younger children will thus favor the information provided by the modality with the highest context-dependent reliability^19,23^. In the case of postural control, children and adolescents until 15 years standing on an oscillating platform displayed better stabilization with open than with closed eyes, thus indicating a strong reliance on vision^3,24^. The display of optic flow patterns to elicit automatic postural movements led to stronger responses in children and adolescents when compared to adults, and the ability to stabilize these movements improved with age until late adolescence^25^. This effect was further enhanced when the participants were standing on a sway-referenced platform^26,27^. When standing on the unstable platform, which attenuates the proprioceptive feedback, adults use primarily vestibular information to stabilize their posture, and this ability matures only during late adolescence^26^.

Interestingly, children aged 7–10 years have been shown to display spatiotemporal muscle activation patterns similar to those observed in adults in response to platform oscillations^28^, revealing an earlier development of automatic postural responses. Similarly, the predominance of visual cues over self-motion has been observed in children up to 11 years in a navigation task^29,30^. The late maturation of visual-vestibular and visual-proprioceptive integration has been correlated with the individual development of these modalities when these are presented in conflict.

The reliance on visual cues can be further challenged by the use of immersive VR, where the participants are immersed in a digital environment through a head-mounted display (HMD). This paradigm led to stronger sensory recalibration^31^ and recruited different adaptation mechanisms^32^ than non-immersive sensory alterations. Thanks to the recent development of lightweight HMDs, the use of VR has expanded to numerous applications designed for children, including neurodevelopmental research^30,33–35^, neurorehabilitation^36–39^, or distraction from painful medical procedures^40,41^. Yet, the majority of these applications offer none or limited interactions with the virtual environment. Therefore, with the exception of two studies showing that children displayed stronger and longer-lasting responses than teenagers to prism adaptation in immersive VR^42^, but generally tolerate this kind of environment^43^, little is known about how children integrate the visual information of the simulated world.

We previously developed a body-machine interface for the immersive control of a first-person view (FPV) flight game in VR relying on simple trunk movements, which was rapidly mastered by healthy adults^44,45^. Here, we adapted this interface to assess the effect of immersive VR on sensorimotor integration and postural strategies in children. Based on the literature review presented above, the transition from ‘en-block’ to segmental upper body coordination occurs between the ages of 6 and 8. We thus first evaluated the ability of children aged 6 to 10 to control the flight game using either their head or their torso, and we assessed the intersegmental coordination patterns which emerged during the execution of this task (Study 1). To further investigate the underlying behaviors, we assessed the head and torso proprioception during a virtual joint angle reproduction task (JAR) with and without explicit visual feedback, in the same age groups (Study 2).

## Results

### Study 1

**Figure 1:**
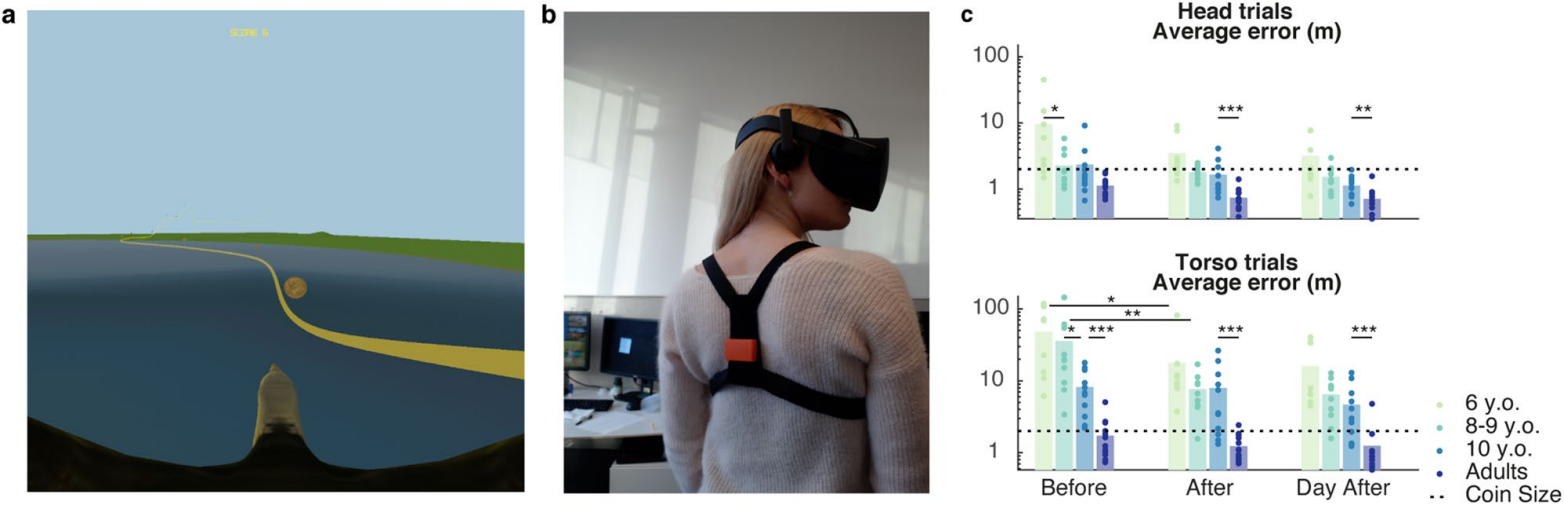
Experimental setup and task performance. (**a**) Virtual environment, as seen by the participant, representing the coins to catch and an underlining ideal trajectory depicted by the yellow line. (**b**) Experimental apparatus worn by the participants, consisting of an HMD and an IMU held in place in the back by a harness. (**c**) Performance on the navigation task, computed as the average distance to the coin centre (error). Dots represent the average error for each individual participant, bars the average across participants. N = 9 (6 y.o.), 12 (8–9 y.o.), 11 (10 y.o.), 13 (adults). Significance levels: *p* < 0.001 (***), *p* < 0.01 (**), *p* < 0.05 (*).

In this study, the participants were asked to play an immersive flight game in VR. They were immersed in a scenario representing a flight on a bird’s back through an HMD, and the goal of the game was to catch a maximum of golden coins aligned along a smooth path in the air (Fig. 1a). Continuous tracking of the head movements enabled a dynamic adaptation of the field of view, allowing the users to look around in the virtual environment (Fig. 1b). The participants’ initial steering ability was evaluated on the first sequence (*Before*), which was followed by two training sequences and a second evaluation (*After*). The stability of their performance was assessed on a final sequence on the next day (*Day After*). The participants were asked to follow this procedure twice, once by controlling the system with their head, and another time by steering the game with their trunk. The latter condition required to decouple vision and steering commands, whereas these aspects were tied in the head-controlled trials (see Supplementary Fig. S1 for a schematic representation).

We expected participants of all ages to be able to steer the flight with their head and reach similar performances, but to find a significant effect of age on the steering ability with the torso, with 10-year-olds approaching adult levels of performance. As we previously observed a clear motor learning effect on healthy adults in performing the same task with an adapted difficulty level, we hypothesized that the younger children would significantly improve their performance with practice. Regarding the coordination pattern and given the novelty of the task, we expected to observe the ‘en-block’ strategy in children aged 6 and 8, with a diminution of this pattern from 10 years onwards.

#### Controlling body part and age affect steering performance

We assessed the steering performance as the average distance to the center of the coins^44,45^ and found a significant effect of Age (F(3,36) = 30.94, *p* < 0.001, η_p_^2^ = 0.721), Control (F(1,36) = 203.90 *p* < 0.001, η_p_^2^ = 0.850) and Phase (F(2,72) = 47.78, *p* < 0.001, η_p_^2^ = 0.570), as well as a significant Age:Control (F(3,36) = 5.88 *p* <= 0.002, η_p_^2^ = 0.329) interaction (Fig. 1c, see also supplementary Fig. S2).

When comparing consecutive age groups in the head-controlled condition, we found a significant difference between the 8- and 6-year-olds *Before* (*p* = 0.025, d = 1.10), and between the 10-year- and the adults *After* (*p* < 0.001, d = 1.72) and on *Day After* (*p* = 0.007, d = 1.27). In the torso-controlled trials, the 10-year-olds outperformed the 8-year-olds *Before* (*p* = 0.014, d = 1.18), and adults surpassed the 10 year-olds and adults in all phases (*Before*: *p* < 0.001, *d* = 2.29; *After*: *p* < 0.001, *d* = 2.07; *Day After*: *p* < 0.001, *d* = 1.83).

At each level of practice, all the age groups reached better scores with their head than with their torso. Lastly, we only observed a learning effect in the torso-controlled condition between *Before* and *After* training for the 6- (*p* = 0.016, d = 0.955) and 8-year-olds (*p* = 0.004, d = 1.20).

#### Segmental coordination and torso involvement differ between torso and head control

**Figure 2:**
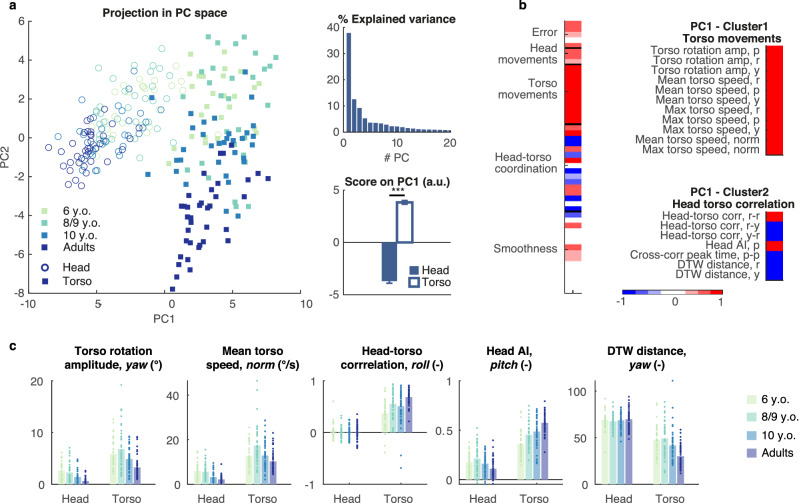
Segmental coordination and torso involvement differ between torso and head trials. (**a**) PCA applied to the data collected on all trials. The projection of the data in the space spanned by the first two PCs displays a control-based separation along the first component (left) representing 37% of the overall variance (top right). This division was confirmed by a t-test (bottom left, mean + SEM). (**b**) Normalized loadings of the descriptive variables on the first PC (left) and variables with absolute loadings higher than a threshold of 0.75 grouped into functional clusters. (**c**) Representative variables selected from the functional clusters with significant effect of Control. Dots represent the average value for each individual participant, bars the average across participants. N = 9 (6 y.o.), 12 (8–9 y.o.), 11 (10 y.o.), 13 (adults).

We next sought to get a global understanding of the behavioral correlates underlying these differences in performance. We applied Principal Component Analysis (PCA) to a set of kinematic variables (see Table 1 for a description of the variables) computed from all trials, and we found that the first principal component (PC) accounted for 37.91% of the dataset's variability and separated the head- from the torso-controlled trials (*p* < 0.001, Fig. 2a). We selected the kinematic variables with normalized loadings > 0.75, which could be clustered into torso movements (Cluster 1) and head–torso coordination (Cluster 2, see Fig. 2b).

**Table 1:** Descriptive kinematic variables.

#	Variable	Details
**Steering performance**		
1–3	Error (a.u.)	Unsigned distance to the coin center, computed when the participant crossed the vertical plane perpendicular to the trajectory supporting the coin
4	Path ratio (–)	Quotient of the travelled path and an ideal path computed as a Catmull–Rom interpolation between the coins. Computed for the entire sequence
5	Time (s)	Duration of the interval between two consecutive coins
**Head movements**		
6–8	Head rotation amplitude (°)	Interquartile range. *Pitch, roll, yaw*
**Torso movements**		
9–11	Torso rotation amplitude (°)	Interquartile range. *Pitch, roll, yaw*
12–14,18	Mean torso speed (°)	Angular velocity. *Pitch, roll, yaw, norm*
15–17, 19	Maximum torso speed (°)	Angular velocity. *Pitch, roll, yaw, norm*
**Head–torso coordination**		
20–24	Head–torso correlation (–)	Absolute correlation. *Pitch-pitch, roll-roll, yaw-yaw, roll-yaw, yaw-roll*
25–27	Head anchoring index (AI,–)	Computed as $\Delta \sigma = \frac{{\sigma_{r} - \sigma_{a} }}{{\sigma_{r} + \sigma_{a} }}$ , where σ_a_ is the standard deviation of the absolute head angles and σ_r_ the standard deviation of the head angles relative to the torso. Positive Δσ values indicate a preferred head stabilization to the external space and negative values a better head stabilization to the torso^9,14^. *Pitch, roll, yaw*
28–32	Peak time of head–torso cross-correlation (s)	Occurrence of the peak in cross-correlation. Negative delays indicate that the head is moving ahead of the body. *Pitch-pitch, roll-roll, yaw-yaw, roll-yaw, yaw-roll*
33–35	DTW distance (a.u.)	Dynamic time warping (DTW) distance between the head and torso sequences. Both segments were linearly interpolated to keep the number of data points constant across sequences^68,69^. *Pitch, roll, yaw*
**Movement smoothness**		
36–38	Torso SAL	3-dimensional smoothness metric based on the arc length of the movement speed profile’s normalized Fourier magnitude spectrum; higher absolute values relate to jerkier movements^70^
39–41	Number of peaks head (–)	Time-normalized number of peaks^71^. *Pitch, roll, yaw*
42–44	Number of peaks torso (–)	Time-normalized number of peaks^71^. *Pitch, roll, yaw*
45–47	Number of peaks bird (–)	Time-normalized number of peaks^71^. *Pitch, roll, yaw*
48–50	Torso speed ratio (–)	Ratio of the mean to the maximum velocities; a ratio close to 1 stands for smooth movements, while lower values indicate jerkier movements^72,73^. *Pitch, roll, yaw*

We found a significant effect of Control on all identified variables. In particular, torso movements were executed with larger yaw amplitude (*p* < 0.001, η_p_^2^ = 0.76) and higher average velocity (*p* < 0.001, η_p_^2^ = 0.87) in the torso-controlled trials (Fig. 2c). Expectedly, head movements were more similar to trunk movements in torso- than in head-controlled trials, as assessed by the head–torso correlation in the roll plane (*p* < 0.001, η_p_^2^ = 0.88) or the dynamic time warp (DTW) distance between both segments in the yaw plane (*p* < 0.001, η_p_^2^ = 0.84). The higher pitch head anchoring index (AI) in the torso-controlled trials (*p* < 0.001, η_p_^2^ = 0.87) reveals that the head is preferentially stabilized to the external space than to the trunk in this condition.

#### ‘En-bloc’ head–torso coordination during torso-controlled trials increases with age

**Figure 3:**
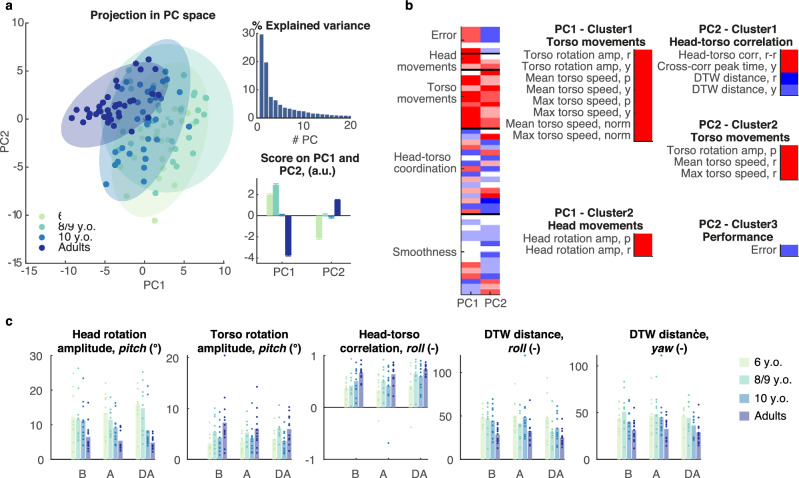
Efficient selection of head–torso coordination strategy develops with age. (**a**) PCA applied to the data collected on torso-controlled trials. The projection of the data in the space spanned by the first two PCs displays an age-based separation along the first two components (left) representing respectively 29.6% and 19.7% of the overall variance (top right). Group means of the scores on the first two PCs (bottom left, mean + SEM). (**b**) Normalized loadings of the descriptive variables on the first PC (left) and variables with absolute loadings higher than a threshold of 0.75 grouped into functional clusters. (**c**) Representative variables selected from the functional clusters with significant effect of Age. Dots represent the average value for each individual participant, bars the average across participants. N = 9 (6 y.o.), 12 (8–9 y.o.), 11 (10 y.o.), 13 (adults). B: Before, A: After, DA: Day After.

To extract the specific variability inherent to torso steering, we repeated the procedure described above, using only the data from the torso-controlled trials. PCA revealed an age-based separation in the space spanned by the first two PCs, accounting respectively for 29.61% and 19.71% of the total variance (Fig. 3a). Individually, both PC1 and PC2 showed a decreasing trend with age (Fig. 3a).

The selection of relevant descriptive variables yielded five functional clusters holding variables describing the torso movements, head movements, head–torso correlation and finally the error (Fig. 3b). All the identified variables showed a significant effect of Age and/or Age:Phase interaction. Younger children displayed larger vertical head movements (*p* < 0.001, η_p_^2^ = 0.38, Fig. 3c) and smaller torso movements (*p* = 0.004, η_p_^2^ = 0.31). Remarkably, the similarity between head and torso movements augmented with age, as revealed by the increased correlation in the roll plane (*p* = 0.014, η_p_^2^ = 0.25) or the DTW distance in the roll (*p* = 0.002, η_p_^2^ = 0.33) and yaw planes (*p* = 0.006, η_p_^2^ = 0.29).

#### Torso involvement in head-controlled trials decreases with age

**Figure 4:**
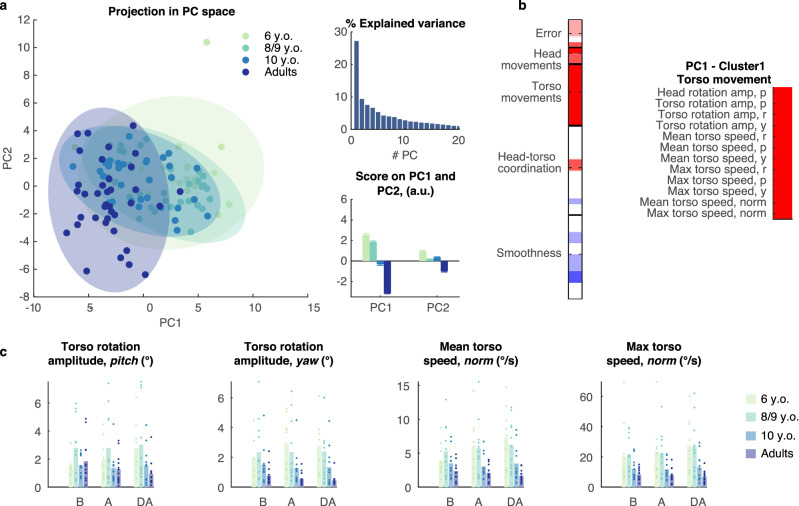
Torso involvement in head-controlled trials decreases with age. (**a**) PCA applied to the data collected on head-controlled trials. The projection of the data in the space spanned by the first two PCs displays an age-based separation along the first component (left) representing 27.2% of the overall variance (top right). Group means of the scores on the first two PCs (bottom right, mean + SEM). (**b**) Normalized loadings of the descriptive variables on the first PC (left) and variables with absolute loadings larger than 0.75 grouped into a functional cluster. (**c**) Representative variables selected from the functional cluster with significant effect of Age. Dots represent the average value for each individual participant, bars the average across participants. N = 9 (6 y.o.), 12 (8–9 y.o.), 11 (10 y.o.), 13 (adults). B: Before, A: After, DA: Day After.

For head-controlled trials, PCA revealed a soft age-based separation along with the first principal component, accounting for 27.2% of the total variance (Fig. 4a). The selected variables all described torso movements (Fig. 4b), and showed a significant effect of Age and/or Age:Phase interaction. The amplitude of the torso movements decreased with age in the pitch (*p* = 0.011, η_p_^2^ = 0.34, Fig. 4c) and yaw planes (*p* < 0.001, η_p_^2^ = 0.37), as well as the average (*p* = 0.002, η_p_^2^ =0.31) and maximal torso velocity (*p* = 0.001, η_p_^2^ = 0.33).

### Study 2

Study 1 revealed an unexpected coordination pattern, where the use of the ‘en-block’ strategy *increased* with age in the torso-controlled condition. To gain a better understanding of the contribution of vision, proprioception, and the effect of the optic flow, we designed a second study consisting of a joint angle reproduction task (JAR). This paradigm is an active test for proprioception, in which the participants are asked to bring a given body part in a predefined orientation. This test reflects the functional use of this sensory pathway and relies on kinaesthetic memory^46,47^, a necessary competence for the proficient use of the flight simulator tested in Study 1.

**Figure 5:**
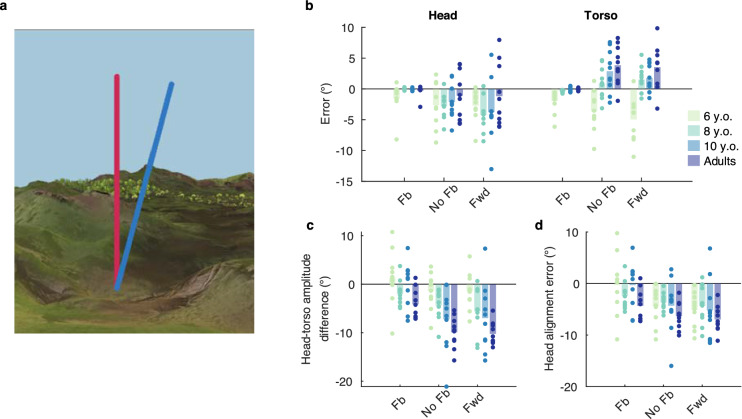
Joint angle reproduction (JAR) test. (**a**) Virtual setting for the Feedback condition. The blue line indicates the target orientation and the pink line the current orientation of the tested body part. (**b**) Signed error at final orientation, positive values indicate final positions exceeding the target angle. (**c**) Difference of head and torso final orientation in the torso JAR, Negative values indicate that the head angle is smaller than the torso angle. (**d**) Difference between final head orientation and target orientation in the torso JAR. Dots represent the average error for each individual participant, bars the average across participants. N = 10 for each age group. Fb: Feedback, No Fb: No feedback, Fwd: Forward, see text for description of the conditions.

As previously, the participants wore an HMD and were immersed in a virtual landscape, to which was superimposed a blue line indicating the target orientation. The participants completed the test under three conditions presented in this order: *Feedback*, where a line indicated the current angle of the tested body part (see Fig. 5a), *No feedback,* where the feedback line was removed, and *Forward*, where a constant forward speed without the feedback line. The task was executed once with the head, and once with the torso.

The *Feedback* condition serves as control block to ensure that the participants are able to execute the task. The *No* feedback condition reveals the maturity of the proprioception. Lastly*,* the addition of a *Forward* speed allows to assess the effect of the optic flow.

We hypothesized that younger children would display larger errors than older participants in the absence of visual feedback, and that this effect would be stronger for the torso than for the head. We also expected the error in the torso JAR without feedback to be predictive of the ability to steer the flight game.

#### JAR error reveals a different maturation stage of head and torso proprioception

We found a significant effect of Age (F(3,35) =15.96, *p* < 0.001, η_p_^2^ = 0.578), Condition (F(2,70) = 280.29, *p* < 0.001, η_p_^2^ = 0.889) and Control (F(1,35) = 7.95, *p* = 0.008, η_p_^2^ = 0.185), and a significant Age:Condition interaction (F(6,70) = 5.24, *p* = 0.01, η_p_^2^ = 0.239).

In the absence of feedback, all age groups except the 6-year-olds significantly increased their error when using their torso compared to the head trials, overshooting the target orientation in the former case and failing to reach it in the latter. The youngest participants in turn failed to reach the target angle with both body parts. (Fig. 5b). Comparing consecutive age groups, we only found a significant difference in the torso JAR between 6- and 8-year -olds in the *No Feedback* (*p* = 0.002, d = − 1.58) and *Forward* conditions (*p* < 0.001, d = − 2.24).

#### Head–torso coordination during torso trials evolves with age

The angular difference between the head and torso orientations at the final position showed a significant effect of Age (F(3,36) = 7.45 *p* < 0.001, η_p_^2^ = 0.383) and Condition (F(2,72) = 32.15, *p* < 0.001, η_p_^2^ = 0.471, Fig. 5c). Finally, there was only an effect of Condition on the alignment error of the head with the target orientation (F(2,72) = 15.601, *p* < 0.001, η_p_^2^ = 0.302, Fig. 5d).

#### Head–torso coordination, but not torso positioning acuity predict the flight game performance

**Figure 6:**
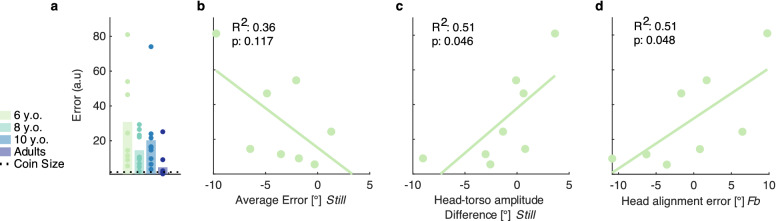
Prediction of flight game steering performance from JAR test. (**a**) Performance during a unique torso-controlled session of the flight game. (**b–d**) Regression analyses on the data of the 6-year-olds, recorded during torso trials of the JAR test. (**b**) Signed error at final orientation (see Fig. 5a). (**c**) Difference between final head orientation and target orientation (see Fig. 5f). (**d**) Difference between final head orientation and target orientation (see Fig. 5g). Dots represent the average error for each individual participant, bars the average across participants. N = 8 (6 y.o.), 10 (8 y.o.), 10 (10 y.o.), 10 (adults). Fb: Feedback, No Fb: No feedback; see text for description of the conditions.

Eventually, we evaluated the relationship between the metrics computed during the JAR test and the performance during one torso-controlled session on the flight simulator (Fig. 6a). For the 6-year-olds, we found a significant relationship of this performance with the head–torso amplitude difference in the absence of visual feedback (*No feedback*: R^2^ = 0.51, *p* = 0.046, Fig. 6c; *Forward*: R^2^ = 0.60, *p* = 0.024), as well as with the head alignment error with *Feedback* (R^2^ = 0.51, *p* = 0.048, Fig. 6d). None of the regressions were significant for the other age groups. Lastly, we found no significant relationship between the torso JAR error and the flight performance (*No feedback:* R^2^ = 0.36, *p* = 0.117, *Forward:* R^2^ = 0.35, *p* = 0.120 for the 6-year-olds, Fig. 6b).

## Discussion

We investigated the development of head–torso coordination when challenged by an alteration of the visual feedback through immersive VR. We first evaluated the ability of children aged 6–10 years and young adults to steer an immersive flight simulator using either their head or their torso (Study 1), followed by a virtual JAR task to further address the behaviors observed during the steering task (Study 2).

All the participants were able to control the flight path using their head in Study 1. A significant difference persisted between all children groups and adults even after practicing the task, but the scores were in a comparable range. When using their torso, 6 and 8-year-olds initially struggled to control the simulator but substantially improved their performance with training. After training, the children’s average error remained higher than the adults'. In the head-controlled trials, the torso involvement decreased with age. Conversely, we observed an increase of the head–torso correlation with age in the torso-controlled trials, thus disproving our original hypothesis.

The virtual JAR test carried out in Study 2 revealed that, in the absence of explicit visual feedback, participants aged 8 and older failed to reach the target angle with their head while exceeding it when performing the task with their torso. The younger children instead failed to reach the desired orientation with both body parts, overestimating their displacement in either case. We also observed that in the torso JAR test, older children and adults decoupled their heads from their torso, maintaining the head close to the vertical during sideward trials. Instead, when explicit feedback was given on the torso position, the 6-year-olds had the tendency to overshoot the target orientation with their head. In addition, we found that for this age group, the amplitude of unnecessary head movements during the torso JAR correlated with their performance in the torso-controlled flight game, while no such relation was found for the amplitude or precision of torso movements.

The comparable performances observed for all age groups in the head-controlled JAR and steering task indicate that children as young as 6 years are able to use and interact with an immersive body-machine interface both for simple and more complex tasks, in line with a recent study^43^. The earlier maturation of the head control is not surprising, as this condition does not require the mastery of an articulated control of the head–trunk unit, which develops from 7 years onwards^10^. However, even in this simpler experimental condition, younger children still display a higher error variability and a larger overshoot, confirming the incomplete development of robust internal models as observed in standard experimental frameworks^2,48,49^.

Kinematic analyses of the head-controlled trials showed that the major age-related difference could be attributed to differences in the torso movements, with rotation amplitudes and mean and maximum rotation velocities are decreasing with age. The ability to decouple head from torso movements thus develops along with childhood, confirming previous results obtained during obstacle avoidance during locomotion^1,15^, where adults display anticipatory head movements^15^. However, mature coordination patterns appear later with our experimental setup when compared to simple locomotion. This is in line with observations revealing that developing children tend to increase their head-body stiffness with increasing task difficulty^9^, and to involve their trunk in situations where such movements are not necessarily required^50,51^. In our case, the increased difficulty can be imputed to the use of immersive VR, which provides altered visual information and requires higher cognitive processing abilities to appropriately interpret the displayed environment^52,53^.

When the control of the flight game was based on torso movements instead, younger children struggled to use the system, even after practicing the task. Assessing the kinematics during this task and the JAR reveals an underlying twofold behavior. First, the age-related increase of the torso amplitude in the steering task and the evolution of the torso JAR error indicate that the immaturity of the torso proprioception leads younger children to overestimate their torso movements. This complements a previous study showing an increase in torso positioning accuracy with age^2^. Second, the larger head movements displayed by the younger participants during the flight game and in the torso JAR with visual feedback suggest that these children attempt to resolve the visual discrepancy by compensatory head movements. This is likely due to the weaker reliability of the neck proprioception, which is not mature yet at this developmental stage^54–56^, and which caused the visual inputs to be weighted more strongly. This behavioral pattern aligns with recent works showing biases in the perception of visual and haptic verticality to unusual body orientations in younger children^57,58^, which is here confirmed by the younger participant’s inability to stabilize their head vertically while aligning their torso to lateral target positions. The stronger reliance on the visual system we observed in younger children has been shown to disappear in adults, where immersive VR appears to increase the contribution of proprioceptive and vestibular inputs to postural control over vision^59^.

The joint display of these two behaviors led to the unexpected observation that only the older participants favorably selected an ‘en-bloc’ strategy with a stiff intersegmental link during the steering task. This opposes the accepted model of postural development, which states that such behavior is preferentially observed in younger children and decreases with age^1,13,15^. One study found a similar behavior in adults, who displayed a head-to-torso stabilization in dimensions in which independent head movements were not beneficial^14^. This is concomitant with our results, as head movements in the torso-controlled trials tended to disturb the participants' spatial orientation. Younger children instead failed to use this simpler pattern, which serves as backup strategy in cognitively challenging conditions at these ages, despite being advised to. While this observation could be imputed to difficulties in following the given indications in the flight game, the consistent observation of this behavior in the simpler JAR task points to an immaturity at the sensorimotor level. This suggests that the altered visual feedback provided by the VR setup strongly reweights the sensory contributions to posture estimation^59,60^ and therefore affects the immature head–trunk coordination by interfering with the default postural pattern.

Immersive VR has the undeniable advantage of creating experimental conditions challenging sensory pathways but comes with certain limitations. The main aspect potentially affecting our study lies in the weight of the HMD, particularly relative to the youngest participants’ head size. While several recent works have used these devices to study sensory integration in developing populations^30,43,61^, none of them specifically addressed this questions. Future studies should evaluate the effect of the added weight on head–torso coordination as function of the participant’s size, for instance by simulating the additional load in an otherwise naturalistic environment. While externally supporting the weight of the HMD without obstructing the participant’s movements seems cumbersome, the foreseen availability of devices with dramatically reduced weights^62^ will allow to further address the question of postural control in immersive VR without this possible confounding factor. Nonetheless, the ‘en-block’ behavior we consistently observed during the head-controlled trials in both studies, which is in line with previous results, as well as the steady shortfall displayed by all participants when using their head in study 2 suggests that this aspect only had a limited impact on these participants.

In this work, we showed that the immersion in a virtual environment where the effects of head and torso are decoupled causes children aged 6 and 8 to deviate from an ‘en-block’ postural control, currently accepted as the default coordination strategy at these ages. This suggests that the still developing proprioception at the neck and torso levels and the strong reliance on visual feedback causes these children to overestimate their torso displacement and to correct the resulting visual discrepancy through compensatory head movements. We argue that, at this developmental stage, the postural control is not yet mature enough to be robust to an alteration of the visual input, which prevents an effective visual-vestibular-proprioceptive sensory integration and confirms that the maturation of motor control extends beyond childhood.

## Methods

### Experimental design

The objectives of the studies presented in this work were to (1) assess the ability of school-aged children to use and interact with an immersive virtual platform, steered by body movements, (2) to compare this ability with the capacity displayed by healthy young adults, (3) identify and describe the coordination patterns which emerge during the use of such a system, (4) evaluate the development of these patterns along childhood, and (5) to disambiguate the contribution of the visual and proprioceptive systems to postural control and motor coordination during the use of the system described in (1).

The study was designed following a repeated measures design, where all participants were asked to use the platform using their head and their torso at multiple timepoints (see *Experimental Protocols* below for details), using the participants’ ages as a between-subjects factor. The participants were randomly assigned to start with their head or their torso. The sample sizes were determined using the software G*power^63^, to reach a significance level of α = 0.05 and a power of (1 − β) = 0.95.

### Subjects

Thirty-six typically developing children participated in the first study, grouped as follows: nine 6-year-olds (5 girls), eight 8-year-olds (2 girls), four 9-year-olds (1 girl) and eleven 10-year-olds (2 girls). Two children (aged 6 and 8) asked to stop the experiment and two other ones (aged 8 and 10) did not comply with the instructions; their data were excluded from further analyses. In addition, 13 healthy adults participated in the study (3 women, age 28.5 ± 3.4 years). Twenty-four typically developing children participated in the second study, grouped as follows: ten 6-year-olds (7 girls), ten 8-year-olds (5 girls), and ten 10-year-olds (5 girls), as well as 10 healthy adults (4 women, age 27.0 ± 3.2 years). Two 6-year-olds did not complete the session with the flight simulator, their data are reported only for the JAR task. None of the children had previously experienced VR, and surveying a representative sample of the participants showed that 86% of them had a tablet at home and 71% regularly played videogames. Both studies were approved by the local ethical committees (Comitato Etico, ASL 3, Genoa, Italy and Commission cantonale d'éthique de la recherche, Geneva Switzerland) and were carried out in accordance with the Helsinki declaration. All the participants or their legal representatives gave their written informed consent to take part in this study. The children were recruited through a network of Genoa (Italy) based schools participating in research projects developed by the Italian Institute of Technology (Genoa, Italy). The children were given a small, low-value gadget for their participation, regardless of their completion of the experiment. The participants were tested between December 2018 and January 2020.

### Experimental setup

The participants were equipped with a head-mounted display (HMD, Oculus Rift) through which they were shown the virtual environment, and an inertial measurement unit (IMU, X-sens MTw Awinda) placed in their back between the scapulae and maintained with a custom harness to acquire their trunk's 3-dimensional (3D) rotation (see Fig. 1b). Custom straps were added to the HMD to hold it in place on the smaller participants’ heads, and the distance between the lenses was adjusted to the participants’ interpupillary distance. The IMU embedded within the HMD was used both to control the view in the virtual environment and to acquire the head rotations. The kinematic data were acquired at a sample period of 68 ms.

### Virtual environment and navigation task

We created a virtual environment (VE) using the game engine Unity3D, which represented a FPV flight on a bird’s back at a constant speed of 12 m/s^44,45^. A succession of coins to catch (distance between consecutive coins: 58m) represented a path to follow, randomly alternating simple forward motion and one of four directional maneuvers (right turn, left turn, ascent, descent). The coins' initial diameter was 1 m, and every time one coin was caught, the next one was enlarged to 2 m. To minimize possible effects of path planning abilities, we additionally displayed a colored line smoothly connecting the coins, computed as a Catmull–Rom spline^64^. Similarly, to provide the participants with a visual cue of their own position in space, an eagle was displayed below their visual horizon (see Fig. 1a). Finally, to keep the experiment engaging, a tinkling sound was played when the coin was caught at a distance smaller than 10 m, which also added points to a total score for the trial, displayed at the top of the screen.

### Control of the flight game

The participants were asked to control the flight simulator using either head or trunk movements. Ascent and descent were achieved by flexion and extension of the controlling body part while right and left turns were computed as a linear combination of lateral flexion and axial rotation. The head and torso rotations were reset to zero before each sequence, at the participants' self-selected neutral position corresponding to a straight, forward flight. Continuous tracking of the head movements also enabled a dynamic adaptation of the field of view, allowing the users to look around in the virtual environment. Steering with torso movements, therefore, required decoupling vision and steering commands, whereas these aspects were tied in the head-controlled trials.

### Experimental protocol study 1

At the beginning of the experimental session, one researcher presented the VE on a computer screen and explained the task. The researcher next demonstrated the control movements and made sure that the participants understood and were able to execute the instructions by asking targeted questions (e.g. “Show me how you would move to turn right/left or to go up/down”). The participants were informed that only movements of the controlling body part (i.e. the head in head-controlled trials and the torso in torso-controlled trials) would have an effect on the trajectory. The experimenter additionally advised them to keep their neck rigid as to move their entire upper body as a whole during the torso-controlled trials, but the choice of the coordination strategy was left open. The subjects were then equipped with the HMD and the IMU, and were seated on a stool or on a chair and asked not to lean against the backrest. The participants were randomly allocated to start the experiment using the head or the torso, using adaptive covariate randomization with the gender as covariate^65^.

The recording sessions took place on two consecutive days. On day 1, the participants had to steer the simulator along four paths with each body part. The first sequence contained 26 coins and was an initial evaluation of the performance (hereafter: *Before*). The second and third sequences each contained 50 coins; these sequences were considered as training. The fourth sequence contained 18 coins (hereafter: *After*). All the sequences controlled with a given body part were executed successively. On day 2, one sequence containing 26 coins had to be performed with each body part (hereafter: *Day After*). Breaks were allowed between the sequences, at the participants' demand.

### Joint angle reproduction (JAR) task

We created a JAR task^46–48^ in virtual reality using the game engine Unity 3D. The participants were immersed in a virtual landscape and were asked to align their head or their torso to one of three predefined orientations (0° and +/− 15°) indicated by a pink line. We tested three conditions: *Feedback*, where a blue line showed the current orientation of the controlling body part, *No feedback*, where the additional visual feedback was removed and *Forward*, where a constant forward speed was simulated. The duration of one trial was set to 4 s, and the participants were asked to hold their final position until the next trial.

### Experimental protocol study 2

At the beginning of the session, one experimenter presented the VE and the control movements ad described above, after which the participants were equipped and seated as in Study 1. The conditions were tested in the following order: *Feedback, No feedback, Forward*, while the participants were randomly allocated to start either with the head or the torso, using covariate adaptive randomization with the gender as covariate^65^. The orientations were presented in a randomized order, totalling 5 repetitions for each orientation in the *Feedback* condition and 10 repetitions for the *No feedback* and *Forward* conditions. At the end of the session, the participants executed one flight sequence with the simulator (*Before* session described above).

### Data processing

The kinematic data acquired in study 1 was divided into segments corresponding to the intervals between consecutive coins. Some segments contained discontinuities due to interferences with the IMU; these segments were automatically identified as consecutive samples displaying angular changes larger than an empirically defined threshold of 20° and rejected for the kinematic analyses. On average, 1 ± 3 segments were rejected from the data collected in Study 1 (82% of the trials without rejection, 3% with more than 10 segments rejected, the latter distributed across age groups) and 2 ± 5 from the data collected in Study 2 (68% of the trials without rejection, 13% with more than 10 segments rejected, the latter distributed across children age groups). Descriptive variables were computed on these segments and averaged over each entire sequence (see Table 1). Principal component analysis (PCA) was applied to the dataset containing the kinematic variables extracted from all trials, or from the head- and torso-controlled trials, respectively. Outliers were detected as data points whose Euclidean distance to the centroid of the z-scored dataset deviated from the average value by more than 4 standard deviations. These points were given a weight of 0.5 in the PCA computation. The variables with normalized loadings > 0.75 on the first (all trials, head-controlled trials) or the first two principal components (torso trials) were considered as significant and were regrouped into functional clusters.

The data acquired during Study 2 was separated into individual trials, and the final position was averaged over the last 1.5 s of each trial. For each trial, we computed the signed error with respect to the target orientation, the overshoot, the number of oscillations around the final angle, and for the trials involving the torso, the head AI (computed over the entire trial), the final angular difference of the head and the torso and the head alignment “error” as the difference between the final head angle and the target orientation.

### Statistical analysis

The normality of the data was tested with the Anderson-Darling test, and a Box-Cox transformation was applied when the test rejected the normality hypothesis. The statistical evaluations were performed with paired (within age groups) or unpaired (between age groups) t-tests or repeated-measures ANOVAs, using the age as a between-subjects factor and the control type and/or experimental phase as within-subject factors using custom Matlab routines^66^. The false discovery rate was controlled using the "two-stage" Benjamini–Krieger–Yekutieli procedure^67^.

## Supplementary Information

Supplementary Figures.

## Data Availability

All data needed to evaluate the conclusions in the paper are present in the paper and/or the Supplementary Materials. Data used for this submission and the processing routines are available at https://github.com/jmlbr/Children_ImmersiveVR.
