# Supplementary material for: Immersive virtual reality interferes with default head–trunk coordination strategies in young children
Source: Sci Rep. 2021 Sep 27;11:17959. doi: 10.1038/s41598-021-96866-8 (PMC8476578; doi:10.1038/s41598-021-96866-8)
Supplement: Supplementary file 1 — Supplementary Figures. [file 41598_2021_96866_MOESM1_ESM.docx]

Supplementary materials for

**Immersive virtual reality interferes with default head-trunk coordination strategies in young children**

Jenifer Miehlbradt, Luigi F. Cuturi, Silvia Zanchi, Monica Gori, Silvestro Micera


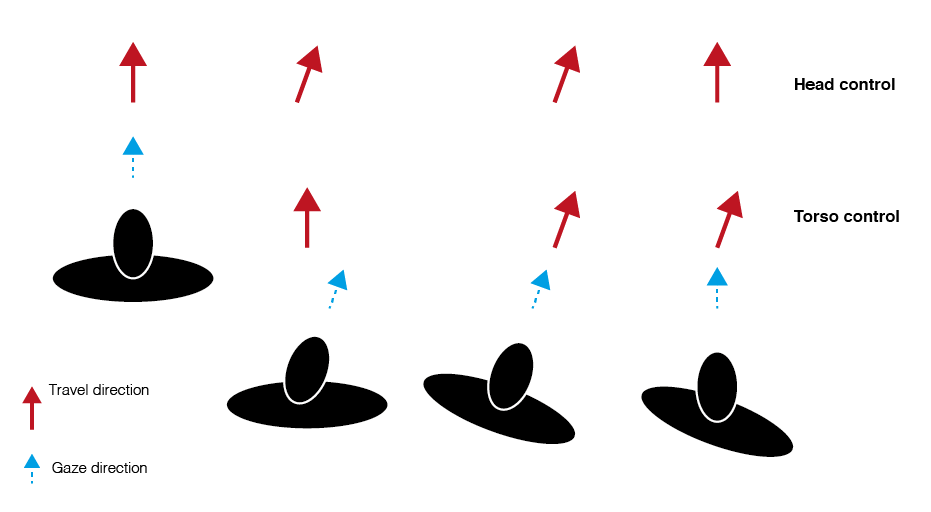


Figure S1 : Effect of head and torso rotations on the gaze and travel directions (above view).

Figure S2:
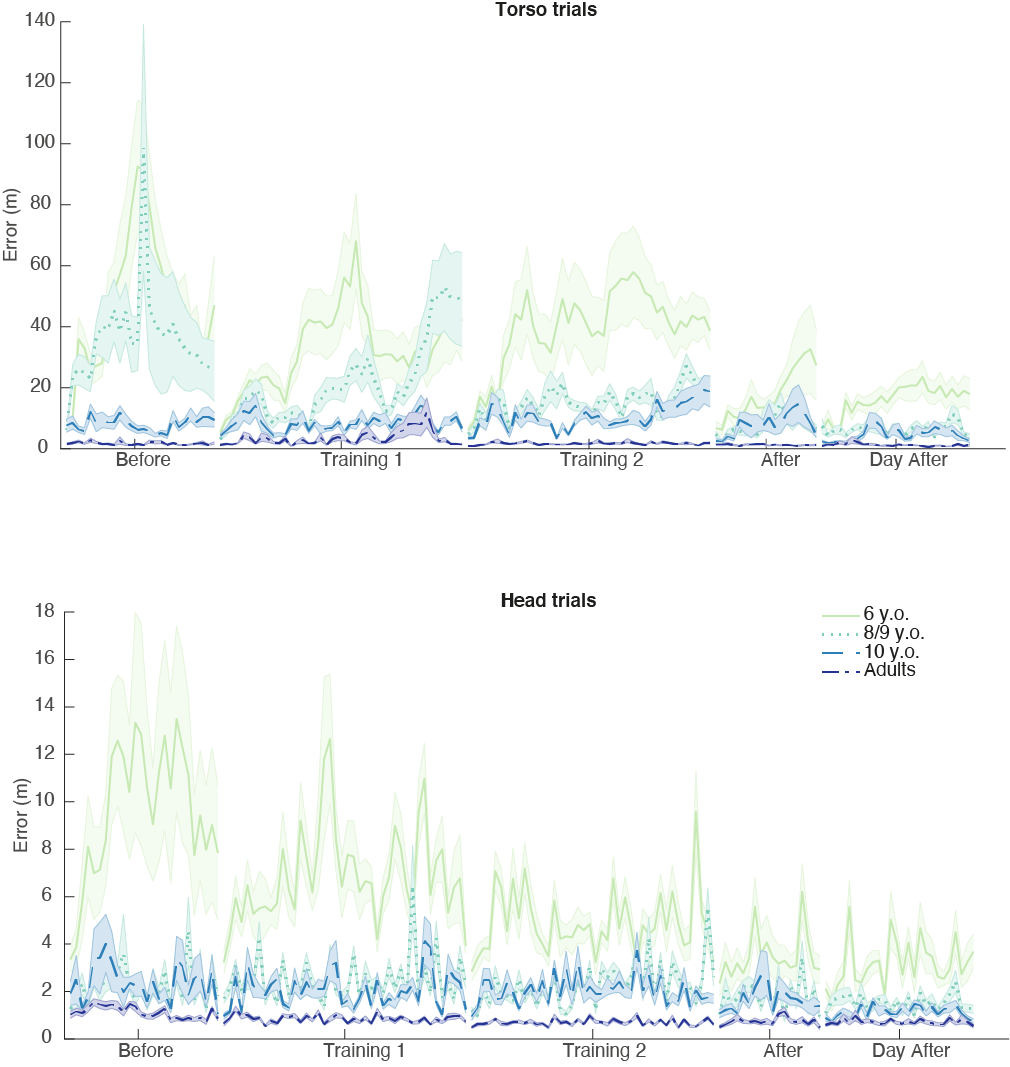
 Raw performance during the flight game (Study 1), by sequence.
